# Mathematical Modeling of Endemic Cholera Transmission

**DOI:** 10.1093/infdis/jiab472

**Published:** 2021-09-22

**Authors:** Dennis L Chao

**Affiliations:** Institute for Disease Modeling; Bill & Melinda Gates Foundation, Seattle, Washington, USA

**Keywords:** Cholera, mathematical modeling, oral cholera vaccine

## Abstract

Mathematical modeling can be used to project the impact of mass vaccination on cholera transmission. Here, we discuss 2 examples for which *indirect protection* from mass vaccination needs to be considered. In the first, we show that nonvaccinees can be protected by mass vaccination campaigns. This additional benefit of indirect protection improves the cost-effectiveness of mass vaccination. In the second, we model the use of mass vaccination to eliminate cholera. In this case, a high population level of immunity, including contributions from infection and vaccination, is required to reach the “herd immunity” threshold needed to stop transmission and achieve elimination.

Oral cholera vaccine (OCV) can be used to combat epidemic and endemic cholera [1]. In some ways, the quantitative support for interventions against endemic cholera, such as vaccination, improved sanitation, and education campaigns, is more straightforward than in nonendemic regions, where outbreaks are sporadic and less predictable. If outbreaks occur regularly, then interventions focus on endemic “hot spots” and are targeted by age or other subpopulation if appropriate. Even with relatively predictable outbreaks, however, estimating the impact of interventions can be difficult. Dynamic modeling, which uses mathematical equations or computer models to simulate disease spread, can capture some of the complicated effects of interventions. Here, we illustrate the use of dynamic modeling to help understand the impact of mass vaccination on endemic cholera transmission in 2 settings: Bangladesh and Haiti.

## DYNAMIC MODELING FOR IMPROVING COST-EFFECTIVENESS ESTIMATES

OCV provides adults good protection for several years, with lower efficacy among young children [2, 3]. However, because young children have generally been found to have higher incidence than adults in endemic settings, it is worth considering prioritizing them for vaccination [4]. To estimate the effectiveness of mass vaccination with OCV, one could start with a simple calculation, where the reduction in cholera cases is simply proportional to vaccine coverage and vaccine efficacy [5]. This could help weigh the merits of prioritizing children (lower efficacy but higher incidence) or adults (higher efficacy but lower incidence) when resources are scarce. However, this calculation ignores the potentially substantial indirect protection from mass vaccination.

**Figure 1.**
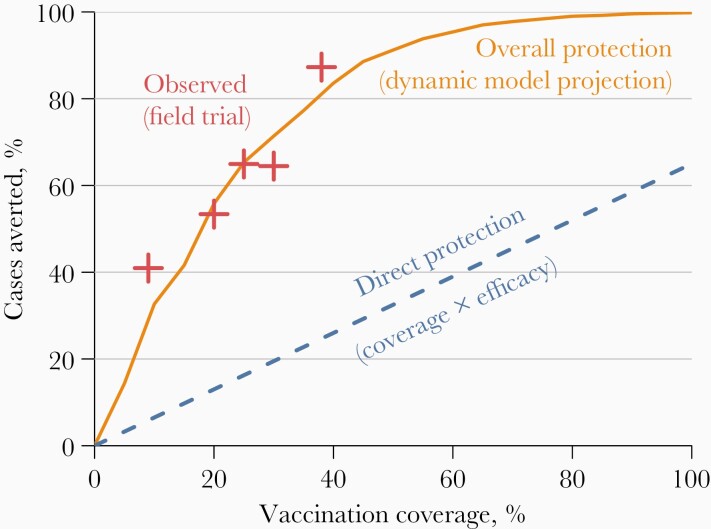
Observed and modeled protection from mass cholera vaccination in Bangladesh. “Direct protection” assumes that vaccine efficacy is 65% and that there are is no indirect protection from vaccination. “Observed” points are based on the field trial described elsewhere [6, 8]. “Overall protection” is derived using the model described by Longini et al [9]. This figure was adapted from Troeger et al [5].

In a reanalysis of an individually randomized OCV trial, cholera incidence was found to be lower among placebo recipients if nearby OCV coverage was high [6]. In other words, nonvaccinees benefited from vaccinated neighbors. This makes intuitive sense—if disease incidence or risk is reduced among your likely contacts, then you are at less risk of infection as well [7]. Figure 1 illustrates the potential magnitude of indirect protection from vaccination. *Direct protection* would protect only those who are vaccinated. The number of cholera cases averted by direct protection is proportional to vaccination coverage, so cholera incidence would be reduced by only about 30%–40%, even with an impossibly high 100% coverage. However, the field trial revealed higher-than-expected protection in areas with high coverage [6], and a dynamic model was later fit to these data to provide a mechanistic interpretation [9]. At sufficiently high levels of vaccination, at which infected individuals infect on average <1 other person, disease transmission basically stops. This phenomenon has been called *herd protection* or *community immunity* [10]. The potentially huge gains from indirect protection became a key part of several cost-effectiveness analyses of OCV [11].

We used dynamic models of cholera transmission to simulate the vaccination of 3 age groups in Dhaka, Bangladesh: 1–4-year-olds, 1–14-year-olds, and everyone ≥1 year old [12, 13]. Briefly, these models used systems of ordinary differential equations to simulate the spread of cholera from infected people or the environment to susceptible people. Transmission from the environment peaked twice each year to capture the seasonal cholera outbreaks in Bangladesh [14]. As expected, vaccinating everyone prevented the most cases in the models, but vaccinating 1–14-year-olds was most cost-effective by preventing the most cases per vaccination. Limiting vaccination to children <5 years old was less effective because vaccine efficacy is lower among young children and because vaccinating a small portion of the population does not substantially reduce overall cholera circulation. Increasing the target to include children up to 14 years old not only directly protected this larger segment of the population, but it also partially protected the rest of the population by reducing cholera transmission from children. In other words, vaccinating children can protect adults.

Because we were evaluating the potential benefit of recurring mass vaccination campaigns, we had to simulate the dynamics of cholera over several years. We assumed that children who were vaccinated before age 5 years would have less protection than those vaccinated when they were older, based on observations reported elsewhere [3]. It was therefore important to revaccinate the youngest children once they were older. The high population coverage that conferred indirect protection was hard to maintain. In addition to vital dynamics (ie, births and deaths) steadily eroding population-level coverage, the high-risk populations living in urban slums had high turnover, making it difficult to maintain sufficient vaccine coverage levels without regular vaccination campaigns to reach newly arrived unvaccinated adults.

## DYNAMIC MODELING OF CHOLERA ELIMINATION

Cholera was introduced to Haiti in late 2010, and hundreds of thousands of cases were reported over the following 2 years [15]. After the first massive waves, cholera appeared to persist at lower levels, with more outbreaks during the rainy season. Unlike in Bangladesh, where *Vibrio cholerae* is an autochthonous part of the Bay of Bengal [16], cholera has not been a persistent presence in the Americas. With no obvious natural reservoir, interrupting transmission among humans could eliminate cholera from Haiti. Interrupting transmission would not require vaccinating every resident of Haiti—vaccines just need to reach enough people to prevent chains of transmission from persisting throughout the year. Dynamic modeling could be used to understand how interventions could stop cholera transmission in the context of seasonality and waning immunity. Several mass vaccination scenarios in Haiti were modeled by 4 independent modeling teams, and the results were reported in [17]. Here, we describe one of those models.

We had adapted a model that was originally developed to understand the potential impact of mass vaccination soon after cholera introduction [18]. Unlike the model for Bangladesh described earlier, the model for Haiti is agent based, meaning each person in the country’s population is represented explicitly. One reason for this computationally expensive modeling choice was to allow us to track the number of cholera cases instead of the proportion of the population that is infected. By tracking the exact number of cases, we can define the “elimination” of cholera to be when the last simulated case of cholera occurs. Simulated individuals reside in an environment that captures a few major geographic features of Haiti, such as roads and proximity to rivers. Individuals living near rivers are exposed to environmental *V. cholerae* from communities living upstream, and individuals living near roads could encounter and be infected by people who live along the same roads. Susceptible children and adults have the same level of exposure to cholera, but adults are more likely to be immune from prior infection. The updated model added waning immunity after cholera infection or vaccination, since we were simulating cholera transmission over several years [17]. We also added seasonality. Cholera transmission appears to be rainfall driven in Haiti, so transmission was higher during Haiti’s rainy season.

**Figure 2.**
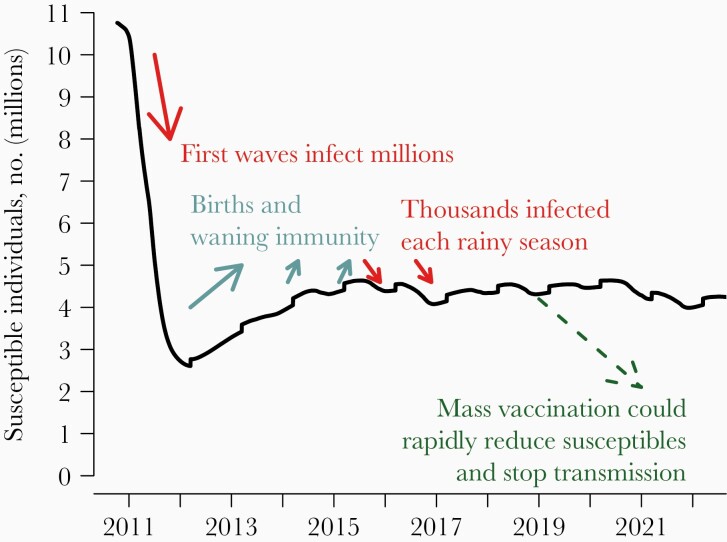
Modeling the dynamics of cholera in Haiti. The panel shows the number of cholera-susceptible individuals in the simulated population from one stochastic run of a model. Millions of people were infected when cholera was first introduced in 2010, causing a steep drop in the number of residents susceptible to cholera. After the initial massive outbreaks, our model predicts that there were annual cycles in the number of susceptible individuals, with the net number increasing owing to births and waning immunity during the dry seasons and decreasing during rainy-season outbreaks. We simulated different mass OCV vaccination strategies starting in January 2019.

Our model found that cholera infected millions of people in Haiti over the first year, which drove down the number of people susceptible to cholera (Figure 2). The high level of population-level immunity from these massive outbreaks blunted outbreaks in subsequent years. This population-level immunity is steadily diminished as (fully susceptible) people are born and immunity wanes among those who had been infected, so the number of susceptible people climbs. When that number is sufficiently high and cholera transmission is facilitated by the annual rainy season, outbreaks occur and drive the number of susceptible persons back down. Because cholera appears to be less transmissible in the dry season in Haiti, there is a chance that the number of cases could drop to zero during a dry season and cholera would disappear from the country [19]. We deliberately calibrated our model to ensure cholera transmission from 2010 to at least 2018 (when the model was developed), which might make the model pessimistic about the possibility of elimination. At the time of writing, the last reported case of cholera in Haiti was in 2019 [20], and it is possible that cholera would or did spontaneously disappear from Haiti without additional interventions.

Driving the number of people susceptible to cholera down below a critical threshold across the country should result in elimination. We found that a rapid mass vaccination campaign covering the whole country within 2 years would have the best chance of eliminating cholera. A slower rollout that spanned 5 years was less effective because it was not fast enough to counter waning vaccine efficacy and vital dynamics—the first department to be vaccinated was able to support cholera transmission by the time the last department was reached. Vaccinating only the most cholera-affected departments was not likely to achieve elimination because cholera could persist in the unvaccinated regions and reinvade the vaccinated departments as immunity waned.

## DISCUSSION

Dynamic models can be used to project the effectiveness of mass vaccination, including indirect or herd protection. For cholera vaccination, where direct protection is moderate but high coverage results in good population-level protection, modeling may be necessary when considering mass cholera vaccination to mitigate or eliminate cholera. We used a dynamic model to show the impact of recurring mass vaccination campaigns in Dhaka over the course of several years, and it was necessary to incorporate vital dynamics and migration, which erode population-level vaccine coverage. We used a different model to simulate the rollout of vaccine in Haiti, which could disrupt the equilibrium between rising susceptibility from waning immunity and vital dynamics and the regular outbreaks that maintain cholera endemicity.

Dynamic models were needed to capture the seasonally varying force of infection and a potential gradual rollout of vaccine across the country. It is more difficult to include the impact of nonpharmaceutical interventions, such as water, sanitation, and hygiene and education campaigns, since it is hard to measure compliance, which could be sensitive to changes in implementation. Therefore, we assumed that mass vaccination reduced cholera transmission below “status quo” levels, which are determined by the effectiveness of other interventions that do not change over time in the model. Similar dynamic models can be used to simulate single outbreaks of epidemic cholera, though parameterization and model features could differ. For example, population dynamics and the seasonality of transmission may be more relevant to endemic cholera dynamics, while changes in behavior due to water, sanitation, and hygiene interventions could have larger effects on outbreaks in populations that are less familiar with cholera [21].

Dynamic models have played an increasingly important role in public health decisions. Measles elimination seemed impossible until an effective vaccine was developed and models quantified the level of vaccination required to prevent transmission and reintroduction [22, 23]. Without modeling, it had seemed obvious to focus influenza vaccination efforts on the elderly, who are at higher risk of severe outcomes, but multiple modeling studies showed that vaccinating schoolchildren would also protect adults, making child vaccination a cost-effective strategy [24–26]. With growing availability of OCV, we believe that modeling will play a key role in planning cholera interventions.

## Notes

***Acknowledgments.*** The author thanks the organizers of the 15th Asian Conference on Diarrhoeal Disease and Nutrition.

***Financial support.*** The author is an employee of the Bill & Melinda Gates Foundation. No funds, grants, or other support was received for this work.

***Potential conflicts of interest.*** Author certifies no potential conflicts of interest. The author has submitted the ICMJE Form for Disclosure of Potential Conflicts of Interest. Conflicts that the editors consider relevant to the content of the manuscript have been disclosed. All authors have submitted the ICMJE Form for Disclosure of Potential Conflicts of Interest. Conflicts that the editors consider relevant to the content of the manuscript have been disclosed.

Presented in part: 15th Asian Conference on Diarrhoeal Disease and Nutrition, Dhaka, Bangladesh. January 28-30, 2020.
